# Double‐bundle posterior cruciate ligament reconstruction procedure leads to better restoration of posterior knee laxity in isolated and multiple ligament knee injuries than single‐bundle procedure

**DOI:** 10.1002/jeo2.70295

**Published:** 2025-06-05

**Authors:** Eiji Kondo, Yoshio Nishida, Zenta Joutoku, Daisuke Kawamura, Koji Iwasaki, Masatake Matsuoka, Tomohiro Onodera, Daisuke Momma, Tomonori Yagi, Norimasa Iwasaki, Kazunori Yasuda

**Affiliations:** ^1^ Centre for Sports Medicine Hokkaido University Hospital Sapporo Japan; ^2^ Department of Orthopaedic Surgery, Faculty of Medicine and Graduate School of Medicine Hokkaido University Sapporo Japan; ^3^ Kawamura Orthopaedic Hospital Furano Japan; ^4^ Knee Research Center, Yagi Orthopaedic Hospital Sapporo Japan

**Keywords:** double‐bundle reconstruction, hamstring tendon, multiple ligament knee injuries, posterior cruciate ligament, posterior knee stability, single‐bundle reconstruction

## Abstract

**Purpose:**

The purpose of the study was to determine whether the knee stability is better with single‐bundle (SB) or double‐bundle (DB) posterior cruciate ligament (PCL) reconstruction. The hypothesis was that DB PCL reconstruction in isolated and multiple ligament knee injuries may be significantly better in the posterior laxity than SB procedure.

**Methods:**

A retrospective study was conducted with 51 patients who underwent PCL reconstruction. Seventeen cases required isolated PCL reconstruction, and the others had the following additional ligament reconstruction; 25 cases required anterior cruciate ligament reconstruction, 11 cases required posteromedial corner reconstruction, and eight cases required posterolateral corner reconstruction. All patients were divided into two groups: In Group S, 20 patients underwent SB PCL reconstruction. In Group D, 31 patients underwent DB PCL reconstruction. Clinical outcomes were evaluated at 2 years or more after surgery. The paired Student *t*‐test, Mann–Whitney *U*‐test and chi‐square test were used to test for significance.

**Results:**

The postoperative anterior‐posterior (AP) translation at 20° and 70° and the relative femur‐tibia position in the anterior and posterior stress radiographs at 90° significantly improved postoperatively in both groups. The postoperative side‐to‐side differences in AP translation at 20° and 70° showed no significant difference between the groups. The relative femur‐tibia position in the posterior stress radiographs at 90° was significantly less (*p* < 0.0001) in Group D (mean, SD, 95% confidence interval; 54.0%, 5.2%, 52.1%–55.8%) than in Group S (43.8%, 5.7%, 41.3%–46.3%). There were no significant differences in the valgus and varus laxities, Lysholm score, International Knee Documentation Committee (IKDC) evaluation, Knee Injury and Osteoarthritis Outcome Score (KOOS), Tegner scale, and complications between the two procedures.

**Conclusions:**

There were no significant differences in the Lysholm score, IKDC evaluation, and KOOS, Tegner scale between both groups although there was significantly better posterior stability in 90° flexion with DB reconstruction.

**Level of Evidence:**

Level III.

**Abbreviations**
ACL: anterior cruciate ligament
AL: anterolateral
AM: anteromedial
BTB: bone‐patella tendon‐bone
CT: computed tomography
DB: double‐bundle
ICRS: International Cartilage Repair Society
IKDC: International Knee Documentation Committee
KOOS: Knee Injury and Osteoarthritis Outcome Score
LCL: lateral collateral ligament
LFC: lateral femoral condyle
LM: lateral meniscus
LTC: lateral tibial condyle
MCL: medial collateral ligament
MFC: medial femoral condyle
MLKIs: multiple ligament knee injuries
MM: medial meniscus
MRI: magnetic resonance imaging
MTC: medial tibial condyle
PCL: posterior cruciate ligament
PF: patellofemoral
PLC: posterolateral corner
PMC: posteromedial corner
SB: single‐bundle

## INTRODUCTION

Posterior cruciate ligament (PCL) injury occurs in isolation, or, more commonly, in conjunction with multiple ligament knee injuries (MLKIs) [1, 20, 21]. The most concomitant injuries in acute PCL tears were posterolateral corner (PLC) and anterior cruciate ligament (ACL).

Several studies have reported favorable results of PCL reconstruction using single‐bundle (SB) techniques. However, the clinical results of this surgery have shown that mild residual posterior laxity is common after surgery [22]. Anatomical studies have shown that the PCL is composed of two functional bundles: the anterolateral (AL) bundle and the posteromedial (PM) bundle [9]. Recently, biomechanical studies demonstrated that double‐bundle (DB) PCL reconstruction provides better stability than SB reconstruction [19, 22, 26]. The choice of grafts is one of the most important issues on reconstructing PCL and/or multiple ligaments. Allogenous tissues may be useful for combined ligament reconstruction, however, they are not always available in every country. Therefore, we have used the autograft as the first choice for ligament reconstructions [6]. Based on these studies, the authors have developed a DB PCL reconstruction procedure with hamstring tendon ‘hybrid’ autografts in isolated and MLKIs [25]. Recent studies have suggested that DB PCL reconstruction procedure presented satisfactory results in both isolated and combined PCL lesions [17], and improved posterior stability of the knee and clinical results compared with SB procedure [15, 22]. On the other hand, no differences in the clinical outcomes between SB and DB PCL reconstructions have been reported [5, 14]. Therefore, the superiority of SB or DB PCL reconstruction remains unproven.

The present study hypothesized that, first, postoperative knee stability may improve after the SB and DB PCL reconstruction procedures in isolated and combined PCL lesions. Second, the DB procedure may be significantly better concerning the posterior laxity than the SB procedure, while there may be no significant differences in the objective and subjective clinical outcomes between the two procedures. The purpose of this study was to test these hypotheses.

## METHODS

### Study design

This retrospective study design using patient data were approved by the institutional review board of the hospital and after obtaining patients' informed consent. (No. 017‐0163). A retrospective, comparative Level III study was conducted at the Hokkaido University Hospital using patients who sustained the unilateral PCL injuries between 2010 and 2020. Two senior orthopaedic surgeons (E.K. and Y.K.), who were sufficiently trained concerning this surgery, performed all operations using the same procedure [6, 25]. After surgery, all patients underwent postoperative management using the same rehabilitation protocol [6, 25]. SB PCL reconstruction using a hamstring tendon autograft was performed during the period between 2010 and 2015 (Group S), and DB PCL reconstruction using two hamstring tendon autografts between 2016 and 2020 (Group D) for all patients who would agree to participate in this study. Each patient showed chronic PCL deficiency at the time of PCL reconstruction surgery. The diagnosis of injured ligaments was made based on a detailed history of the knee injury, physical examination on pathologic status and abnormal laxity, routinely performed plain radiographs and magnetic resonance imaging (MRI) scans, and the findings at the time of surgery. In addition, patients with any previous operations for ligament injuries, a concurrent fracture, or osteoarthritis (Kellgren and Lawrence classification > grade 2), were excluded. The time from onset of injury to reconstruction surgery was 3 months or more. All the patients were clinically and radiologically evaluated at least 2 years or more after surgery.

### Preoperative examination

After administration, radiographic evaluations, including MRI and computed tomography (CT) were performed as soon as possible to determine surgical strategies. Then, anterior, posterior, valgus, and varus stress tests were performed. CT angiography was performed if the patient showed any suspected signs of popliteal arterial injury.

### Surgical procedure

Manual reduction was performed if the patient showed knee dislocation. One and two cases required manual reduction in Groups S and D, respectively. Each patient underwent diagnostic arthroscopy immediately before knee surgery. Diagnostic arthroscopy was performed with standard AL and anteromedial (AM) parapatellar portals to confirm that there was PCL injury. Meniscal and chondral lesions were treated arthroscopically if observed.

### SB PCL reconstruction

For SB PCL reconstruction, the semitendinosus and gracilis tendons of the ipsilateral or contralateral knees were harvested using a tendon stripper. Graft choice strategy in isolated PCL injuries and MLKIs were shown in Tables 1 and 2. A pair of the semitendinosus and gracilis tendons was used for PCL reconstruction [6, 10, 25]. Then, the tendons were fashioned so that the graft was comprised of six strands of tendons having a minimum of 70 mm in length and 9 mm in diameter. A 10‐mm wide Leeds–Keio polyester tape (Neoligament) was connected around each end of the tendon loop.

**Table 1:** Graft choice in chronic stage reconstruction.

	ACL	PCL	PMC	PLC
PCL	N/A	Ipsilateral STG	N/A	N/A
PCL/PMC	N/A	Ipsilateral STG	Contralateral ST	N/A
PCL/PLC	N/A	Ipsilateral STG	N/A	Ipsilateral biceps femoris tendon
PCL/ACL	Ipsilateral STG	Contralateral STG	N/A	N/A
PCL/ACL/PMC	Ipsilateral BTB	Ipsilateral STG	Contralateral ST	N/A
PCL/ACL/PLC	Ipsilateral STG	Contralateral STG	N/A	Ipsilateral biceps femoris tendon

Abbreviations: ACL, anterior cruciate ligament; BTB, bone patellar tendon bone; N/A, not available; PCL, posterior cruciate ligament; PLC, posterolateral corner; PMC, posteromedial corner; ST, semitendinosus tendon; STG, semitendinosus and gracilis tendons.

**Table 2:** Patient demographics.

Variable	All injured knee (*n* = 51)	Group S (*n* = 20)	Group D (*n* = 31)	*p*‐Value
Sex, male: female, No.	44: 7	14: 6	30: 1	0.0110
Age, years	34.6 (12.6) (range, 16–61)	36.6 (13.2)	33.4 (12.0)	0.4125
Height, cm	170.8 (5.6)	168.8 (6.5)	172.1 (4.6)	0.0769
Body weight, kg	72.8 (13.1)	73.3 (15.1)	72.4 (11.8)	0.9009
BMI, kg/m^2^	24.9 (4.6)	25.8 (5.5)	24.4 (3.8)	0.4330
Damaged ligaments, No.				
PCL	17	7	10	0.6695
PCL, PMC	5	2	3	
PCL, PLC	4	2	2	
PCL, ACL	13	5	8	
PCL, ACL, PMC	8	4	4	
PCL, ACL, PLC	4	0	4	
Surgical procedure, No.				
SB/DB PCL reconstruction	20/31	20/0	0/31	0.6143
SB/DB ACL reconstruction	17/8	9/0	8/8	
PMC reconstruction	11	4	7	
PLC reconstruction	8	2	6	
PCL graft choice, No.				
Ipsilateral STG	34	15	19	0.3106
Contralateral STG	17	5	12	
Associated injury, No.				
MM: Resection/Suture	2/2	0/0	2/2	0.1453
LM: Resection/Suture	0/3	0/1	0/2	>0.9999
Cartilage injury				
ICRS Grade I	3	1 (LFC)	2 (MTC, LTC)	>0.9999
Grade II	4	2 (MFC, PF)	2 (MFC)	0.6403
Grade III	2	0	2 (MFC, MTC)	0.5137
Grade IV	0	0	0	>0.9999
Peroneal nerve injury	1	0	1	>0.9999
Injury‐to‐operation interval, mo	22.5 (45.2)	21.1 (48.7)	23.4 (42.8)	0.6156

*Note*: Data are reported as mean ± SD unless otherwise indicated.

Abbreviations: ACL, anterior cruciate ligament; BMI, body mass index; DB, double‐bundle; ICRS, International Cartilage Repair Society; LFC, lateral femoral condyle; LM, lateral meniscus; LTC, lateral tibial condyle; MFC, medial femoral condyle; MM, medial meniscus; MTC, medial tibial condyle; PCL, posterior cruciate ligament; PF, patellofemoral joint; PLC, posterolateral corner; PMC, posteromedial corner; SB, single‐bundle; STG, semitendinosus and gracilis tendons.

**Figure 1:**
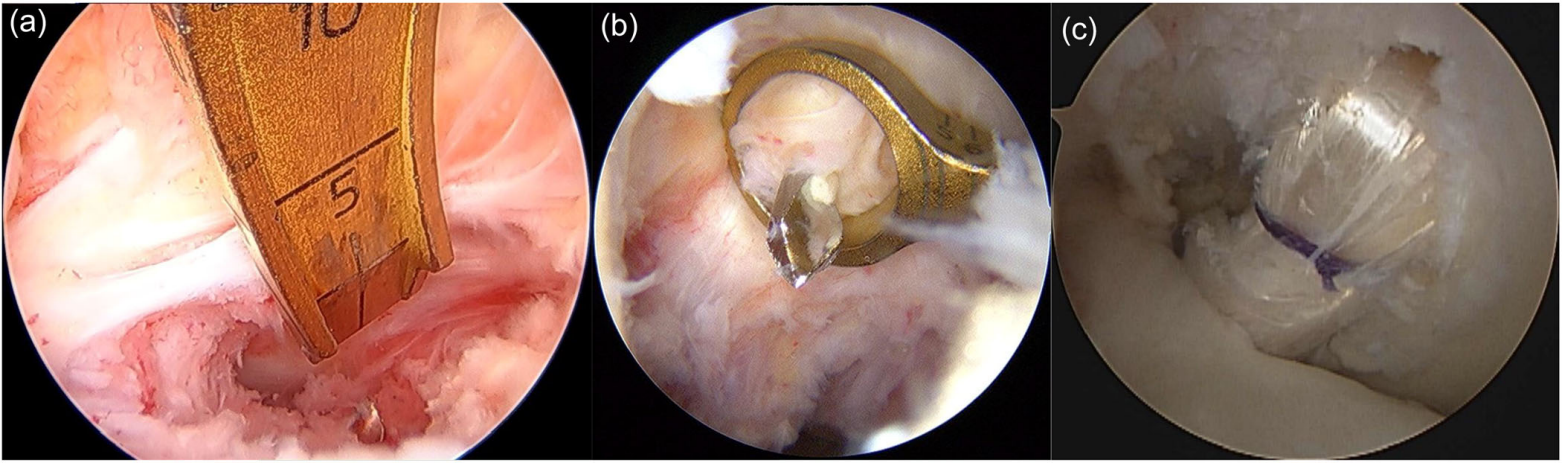
Single‐bundle posterior cruciate ligament (PCL) reconstruction procedure. (a) Arthroscopic view from the anterolateral portal showed tibial tunnel creation using a tibial drill guide. (b) Anterolateral bundle tunnel creation using the outside‐in guide. (c) Single‐bundle PCL graft.

Tibial and femoral tunnels for SB PCL reconstruction were created according to the previous studies [6, 10]. The intraarticular tip of the tibial drill guide (ACUFEX Director PCL Tibial Aimer, Smith & Nephew) was fixed at the centre of the AL bundle attachment on the posterior cortex of the tibia (Figure 1a). After a guidewire was inserted, the tibial tunnel was created using a cannulated drill with a diameter matching the width of the prepared graft. Then, the femoral tunnel was created using the outside‐in technique. A guidewire was inserted using the femoral drill guide (ACUFEX Director PCL Femoral Aimer, Smith & Nephew) (Figure 1b). The femoral tunnel was created using a cannulated drill.

Regarding graft passage, the hybrid graft for SB PCL reconstruction was placed through the tibial tunnel into the femoral tunnel. The femoral side of the tape portion of the PCL graft was then first fixed with two spiked staples (Smith & Nephew). The graft for the PCL was tensioned at 90° of flexion by applying an anterior drawer force to obtain a proper anatomic position in comparison with the contralateral knee (Figure 1c). The knee was then positioned at 10° of flexion through a sufficient tensioning of the PCL graft. A surgeon simultaneously secured PCL grafts onto the AM aspect of the proximal tibia with two spiked staples.

### DB PCL reconstruction

For DB PCL reconstruction, one of the half semitendinosus and gracilis tendons were doubled (diameter: 8–9 mm, length >70 mm), and a 10‐mm wide Leeds–Keio polyester tape (Neoligament) was connected at each end in the same manner as the SB PCL graft. This graft was used for the AL bundle graft of DB PCL reconstruction. The remaining half semitendinosus tendon was also doubled (diameter: 6–7 mm, length >70 mm) with a polyester tape being connected at each end in the same manner. This graft was used for the PM bundle graft.

**Figure 2:**
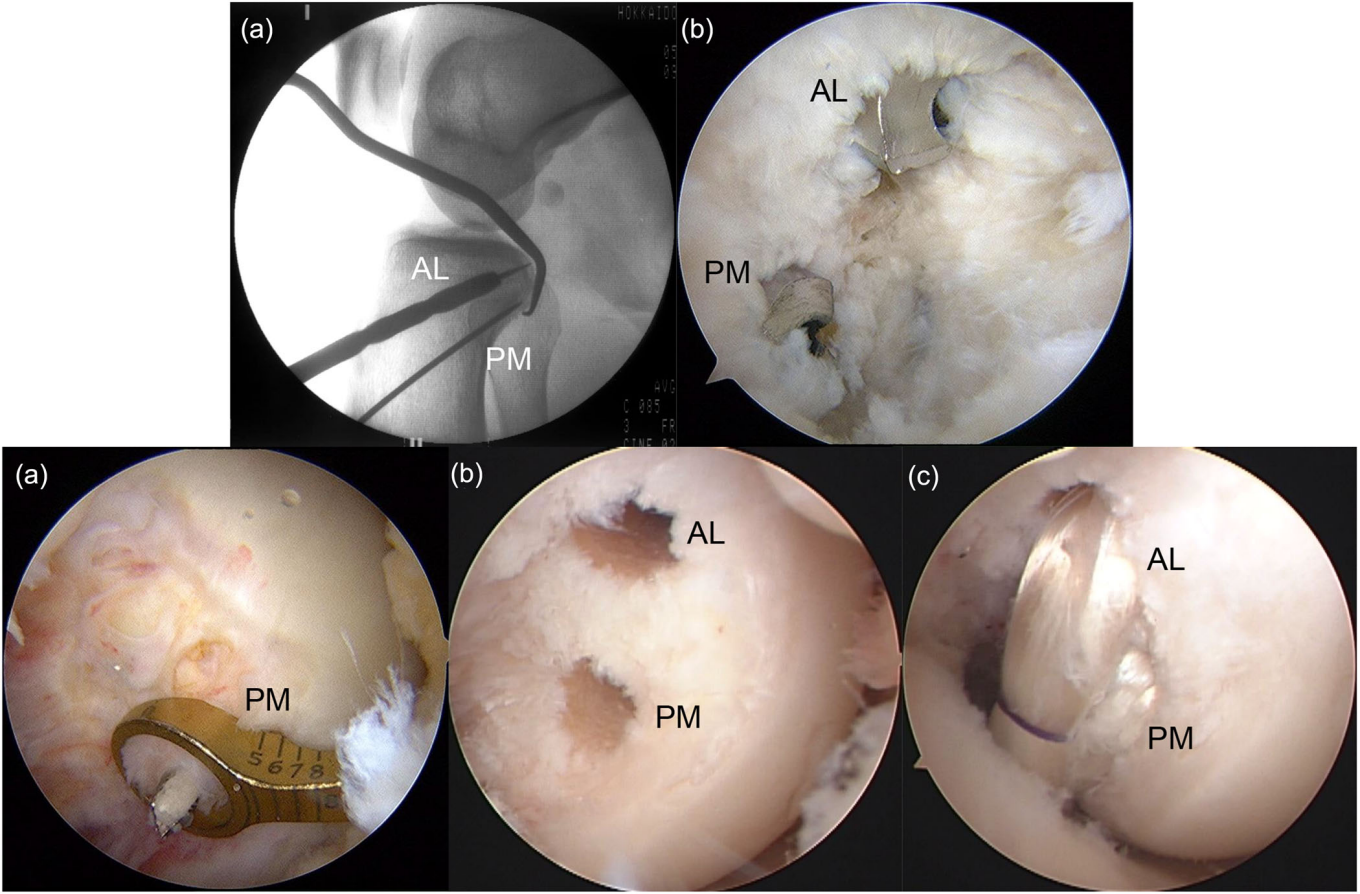
Double‐bundle posterior cruciate ligament (PCL) reconstruction. (a) Tibial tunnel creation using intraoperative fluoroscopy. (b) Arthroscopic view from the posteromedial portal showed two tibial tunnels. (c) Arthroscopic view from the anterolateral portal showed the posteromedial femoral tunnel creation using an outside‐in guide. (d) Two femoral tunnels. (e) Anterolateral and posteromedial bundle hybrid grafts. AL, anterolateral; PM, posteromedial.

The AL and PM tunnels for PCL reconstruction were created according to previous studies [13, 25]. First, the intraarticular tip of the tibial drill guide (Smith & Nephew) was fixed at the centre of the PM bundle attachment on the posterior cortex of the tibia. A guidewire was inserted into the tibia. Next, the intraarticular tip of the guide was fixed to the centre of the AL bundle attachment on the posterior tibia. A guidewire was inserted in the same manner (Figure 2a). The PM and AL tibial tunnels were created using a cannulated drill (Figure 2b). Then, two femoral tunnels were created using the outside‐in technique. After two guidewires were inserted (Figure 2c), the AL and PM femoral tunnels were created using a cannulated drill (Figure 2d).

Regarding graft placement, first, the PM bundle hybrid graft was passed through the PM tibial tunnel into the PM femoral tunnel. The AL bundle hybrid graft was passed in the same manner. The femoral side of the tape portions of the two PCL grafts were fixed first with two spiked staples. The two PCL grafts were fixed onto the AM aspect of the tibia in the same manner as the SB PCL graft (Figure 2e).

### Other ligament reconstruction

In the chronic cases, patients were simultaneously reconstructed for all injured ligaments which had grade III instabilities. In principle, the authors selected an appropriate ipsilateral or contralateral autogenous tendon graft from the semitendinosus and gracilis tendons, the bone‐patella tendon‐bone (BTB), or the biceps femoris tendon for each injured ligament (Table 1) [10].

### ACL reconstruction

ACL reconstruction was performed using the trans‐tibial tunnel technique [25]. First, the tibial tunnel was created. To insert a guidewire, a hole‐in‐one guide (Wire‐navigator, Smith & Nephew) was used. The intraarticular tip of the tibial indicator was placed at the tibial attachment. The guidewire was over‐reamed with a cannulated reamer to the measured graft size. The femoral tunnel of the ACL was created through the tibial tunnel at the point of the femoral attachment using 5‐mm off‐set guide (transtibial femoral ACL drill guide, Arthrex, Naples, FL). After a guidewire was inserted, the femoral bone socket was enlarged to the measured graft size.

### Posteromedial corner (PMC) reconstruction

PMC reconstruction was performed using the previously reported superficial medial collateral ligament (MCL) reconstruction [12]. Briefly, the semitendinosus tendon was doubled. Then, a commercially available polyester tape (Leeds–Keio polyester tape) and (ULTRABUTTON Adjustable Fixation Device, Smith & Nephew) were connected through the tendon loop. The length of the tendon portion of the hybrid graft was approximately 100–120 mm, and the diameter was 6–7 mm.

For PMC reconstruction, a guidewire was drilled at the center of the femoral attachment of the superficial MCL on the medial femoral condyle (MFC). Special care was taken to avoid penetrating this guidewire through the femoral tunnels previously created for cruciate ligament reconstruction. This guidewire was over‐reamed with a cannulated drill, which had a diameter that matched the graft's diameter. To create a tibial tunnel, a guidewire was drilled at a distal point of the tibial attachment of the superficial MCL. The tibial guidewire was over‐reamed with a cannulated drill.

After each graft for the cruciate ligament reconstruction was placed into the femoral and tibial tunnels and fixed at the femoral side. The hybrid graft for PMC reconstruction was implanted into the femoral tunnel. The tibial tape portion of the graft was passed through the tibial tunnel. All the tibial ends of the grafts were tensioned with manual maximum forces and fixed together onto the tibia at 30° of flexion with two spiked staples avoiding the valgus position.

### PLC reconstruction

For the PLC reconstruction, the biceps femoris tendon graft was used as the modified Clancy method according to the previous studies [6, 13]. After the iliotibial band was retracted anteriorly, the peroneal nerve was carefully released from the proximal fibula and the posterior of the biceps femoris tendon. The anterior half of the biceps femoris tendon was released from the proximal portion. Then, the biceps femoris tendon graft was created. An adjustable cortical fixation system (Smith & Nephew) was rigidly connected to the proximal portion of the biceps femoris tendon graft. A guidewire was inserted from the proximal attachment of the lateral collateral ligament (LCL). It is essential that the surgeon must avoid the tunnel convergence to the femoral ACL tunnel due to overlapping [10]. The femoral socket was created to the graft diameter using a cannulated drill. After the graft for cruciate ligament reconstruction was placed into the femoral and tibial tunnels and fixed at the femoral side, the graft for PLC reconstruction was implanted into the femoral tunnel. The graft for the PLC reconstruction was tensioned at 30° of flexion for the final graft fixation avoiding the varus position.

### Postoperative management

Postoperative management was performed according to the previous rehabilitation protocol [10, 25]. Rehabilitation included knee brace immobilization at 10° of flexion for 2 weeks. Partial weight bearing was permitted after 2 weeks after surgery. Full weight bearing was allowed postoperatively after 4 weeks. A long leg hinged dynamic knee brace was used for prevention of posterior subluxation until 3 months after surgery. After 12 weeks postoperatively, several types of athletic exercise (strength and balance training, cycling) were gradually allowed, although no running was allowed until 6 months after surgery. Return to full sports activity was generally permitted at 12 months after surgery.

### Clinical evaluation

**Figure 3:**
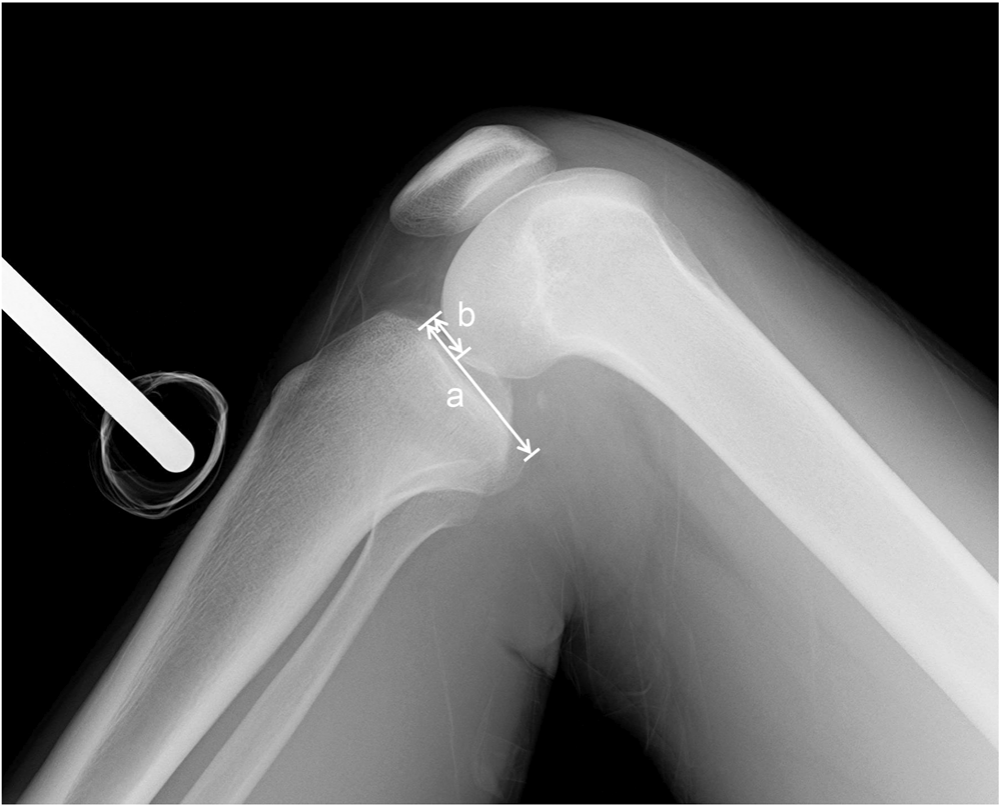
Evaluation of the femur‐tibia position on stress radiograph in 90° of knee flexion. A line was drawn along the tibial plateau, and b line parallel to the tibial plateau were drawn from the anterior of tibial plateau to the overlapped medial and lateral femoral posterior condyles. The ratio of the length from the anterior of tibial plateau to the midpoint of femoral posterior condyles and anterior to the posterior of tibial plateau was indicated as a percentage.

Each patient underwent clinical examinations at least 2 years or more after surgery. One experienced orthopaedic surgeon who was not involved with the surgery examined the patients and evaluated their knee function. Anteroposterior (AP) knee laxity was examined with the KT‐2000 arthrometer (MEDmetric) and stress radiography. Total AP displacement with an applied anterior or posterior force of 133 N was measured at 20° and 70° of flexion with the KT‐2000 arthrometer. Stress radiographs were obtained in 90° of flexion with an applied anterior or posterior force of 133 N with a Stryker knee laxity tester (Stryker Corporation) (Figure 3) [6, 10]. The tibial displacement relative to the femur was measured on the lateral radiographs. The ratio of the length from the anterior of the tibial plateau to the midpoint of femoral posterior condyles and anterior to the posterior of tibial plateau was indicated as a percentage [6, 10]. To assess objective varus and valgus instabilities, a stress radiograph examination was performed under varus and valgus stress with the knee at 20° of flexion using the Telos device (Gmbh, Hungen/Obbornhafen). The degree of total joint space opening was calculated according to the method previously reported [12]. Concerning the International Knee Documentation Committee (IKDC) objective evaluation, the degree of joint line opening was indicated a delta compared to the contralateral side [12]. Peak isokinetic torque of the quadriceps and hamstrings were measured at an angular velocity of 60 degree/s using Cybex II (Lumex) in both knees after surgery. Mean muscle torque, as measured three times postoperatively in the involved knee, is presented as a percentage of the uninvolved knee's value. The Lysholm score, the objective IKDC evaluation form, and Knee Injury and Osteoarthritis Outcome Score (KOOS) were used to evaluate postoperative knee function. The activity levels before injury and at the follow‐up period were also evaluated using the Tegner activity scale. The preoperative values of each clinical score were assessed from the electronic medical record system.

### Statistical analysis

All data are presented as means ± standard deviation. An a priori power analysis was performed. Based on previous studies [15, 22, 26], a sample size of 51 patients (51 knees) was calculated to have 80% power to test the hypothesis. Data were assessed for normality using the Shapiro–Wilk test. The paired Student *t* test was used to assess the difference between the injured and uninjured knees, and pre‐ and postoperative values. The Mann–Whitney *U* test and the chi‐square test were used to test for significance between the two groups. The significance level was set at *p* = 0.05. All statistical analyses of the data were performed using statistical software (GraphPad Software).

## RESULTS

### Patients' demographics

**Figure 4:**
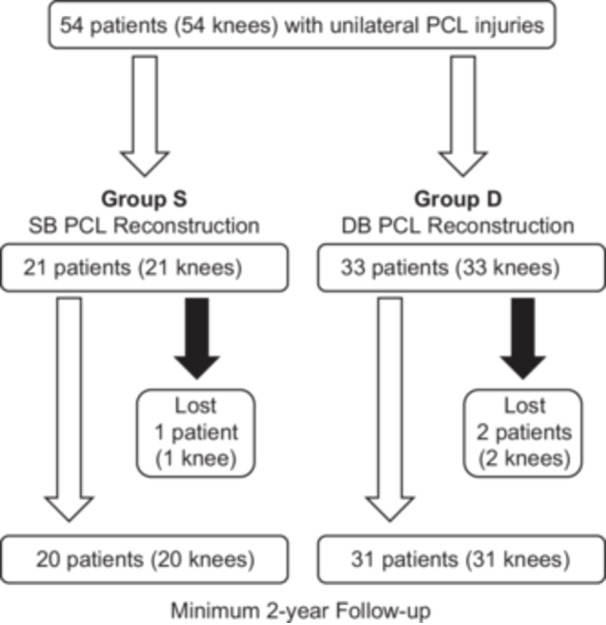
Flowchart of patient inclusion in this study. DB, double‐bundle; PCL, posterior cruciate ligament; SB, single‐bundle.

All 54 initial consecutive patients (54 knees) who sustained PCL injuries were enrolled in this study (Figure 4). Some patients (*n* = 1 in Group S, and *n* = 2 in Group D) were excluded because they were lost to follow‐up. These patients were excluded from the analysis.

Ultimately, 51 patients with a mean age of 34.6 years were registered for evaluations (Table 2). Seventeen cases required isolated PCL reconstruction, and the others had the following additional ligament reconstruction associated with PCL reconstruction according to the previous reported techniques [6, 10, 12, 13, 25], 25 cases required ACL reconstruction, 11 cases PMC reconstruction, and 8 cases required PLC reconstruction. In Groups S and D, there were 20 knees and 31 knees, respectively. The follow‐up period averaged 31.8 months (range, 24–42 months). The cause of injury included 16 motor vehicle accidents, 24 sports‐related injuries and 11 work‐related injuries.

The surgical strategy for MLKIs was as follows [6, 10], in the acute cases (<3 weeks after injury), the repair of the grade III PMC or PLC injuries were performed in the first stage. Simultaneous PCL and/or ACL reconstruction were performed in the second stage [25]. In chronic cases (>3 weeks after injury), a single‐stage PCL and/or ACL reconstruction and/or collateral ligament reconstruction [12, 13] were performed. In the acute cases, four knees required PMC repair and one required PLC repair at the first‐stage surgery [7]. Simultaneous PCL and/or ACL reconstructions were performed at the second stage. One knee that underwent an initial PMC repair had residual valgus laxity and underwent a PMC reconstruction at the time of the PCL reconstruction. In the chronic cases, 25 knees underwent ACL reconstructions, 11 knees underwent PMC reconstructions and eight knees underwent PLC reconstructions at the time of surgeries (Table 2). Preoperatively, complete peroneal nerve palsy was identified in one patient in Group D. There were no statistical differences in background factors between the two groups expect the gender difference.

### Additional treatment

Meniscus and cartilage treatments were shown in Table 2. Debridement was performed for fragmentation of the cartilage in two knees and four knees in Groups S and D, respectively. No treatment was administrated for softening or fissuring of the articular cartilage.

### Knee stability

The side‐to‐side difference in the total AP translation at 20° and 70° significantly improved postoperatively in both groups (*p* < 0.0001) (Table 3). There were no significant differences in the postoperative AP translation at 20° and 70° between the groups. Concerning the isolated PCL injuries, the postoperative AP translation at 20° and 70° was less in Group D than in Group S (Table 4).

**Table 3:** Knee stability.

	All injured knee Pre	All injured knee Post	Group S (*n* = 20) Pre	Group S Post	Group D (*n* = 31) Pre	Group D Post	*p*‐Value Pre vs. Post in Group S	Pre vs. post in Group D	Intragroup comparison
AP laxity, mm	5.5	2.1	5.6	1.9	5.3	2.2	<0.0001	<0.0001	0.7358
20°	(2.4, 4.8–6.1)	(1.7, 1.6–2.6)	(2.0, 4.7–6.5)	(2.2, 1.0–2.9)	(2.6, 4.4–6.2)	(1.3, 1.7–2.6)			
70°	7.1	2.4	7.3	2.7	6.9	2.0	<0.0001	<0.0001	0.6134
	(1.8, 6.6–7.5)	(2.2, 1.7–3.0)	(1.9, 6.4–8.1)	(2.1, 1.8–3.7)	(1.7, 6.3–7.5)	(2.3, 1.2–2.8)			
Anterior drawer, %	65.9	62.3	65.9	62.5	65.8	62.1	0.0248	0.0335	0.9817
	(5.1, 64.5–67.3)	(4.9, 60.9–63.7)	(5.1, 63.7–68.2)	(2.3, 61.5–63.6)	(5.0, 64.1–67.6)	(6.1, 60.0–64.3)			
Posterior drawer, %	37.1	49.5	34.9	43.8	38.2	54.0	0.0030	<0.0001	<0.0001
	(8.2, 34.9–39.3)	(7.4, 47.5–51.6)	(7.8, 31.5–38.3)	(5.7, 41.3–46.3)	(8.1, 35.3–41.0)	(5.2, 52.1–55.8)			
Medial opening, mm	9.4	7.5	9.0	7.4	9.6	7.6	0.0025	<0.0001	0.3345
	(1.8, 8.9–9.9)	(1.0, 7.2–7.8)	(1.6, 8.3–9.7)	(1.1, 6.9–7.8)	(1.9, 8.9–10.2)	(1.0, 7.2–7.9)			
IKDC, No.							0.0644	0.0048	0.9698
A	29	46	12	18	17	28			
B	14	5	5	2	9	3			
C	8	0	3	0	5	0			
D	0	0	0	0	0	0			
Lateral opening, mm	10.6	9.2	10.4	9.2	10.7	9.2	0.0702	0.0042	0.8670
	(2.3, 9.9–11.2)	(1.4, 8.8–9.6)	(2.2, 9.4–11.4)	(1.3, 8.6–9.7)	(2.3, 9.9–11.5)	(1.5, 8.7–9.7)			
IKDC, No.							0.1450	0.0355	0.9138
A	29	43	12	17	17	26			
B	14	8	6	3	8	5			
C	5	0	2	0	3	0			
D	3	0	0	0	3	0			

*Note*: Data are reported as mean (SD, 95% confidence interval) unless otherwise indicated. Medial and lateral joint opening: IKDC grade; A (0–2 mm), B (3–5 mm), C (6–10 mm). D (>10 mm).

Abbreviations: AP, anteroposterior; IKDC, International Knee Documentation Committee.

**Table 4:** Knee stability in isolated posterior cruciate ligament injuries.

	All injured knee Pre	All injured knee Post	Group S (*n* = 7) Pre	Group S Post	Group D (*n* = 10) Pre	Group D Post	*p*‐Value pre versus post in Group S	Pre versus post in Group D	Intragroup comparison
AP laxity, mm	5.0	1.6	5.5	1.8	4.5	1.4	<0.0001	<0.0001	0.7358
	(1.6, 4.2–5.7)	(1.8, 0.7–2.4)	(1.2, 4.6–6.4)	(2.2, 0.2–3.4)	(1.8, 3.4–5.7)	(1.5, 0.5–2.3)			
20°	7.8	2.0	8.6	2.1	7.3	1.9	<0.0001	<0.0001	0.6134
70°	(1.6, 7.0–8.6)	(1.8, 1.1–2.8)	(1.3, 7.6–9.5)	(2.1, 0.5–3.7)	(1.7, 6.2–8.3)	(1.4, 1.0–2.7)			
Posterior drawer, %	36.2	50.6	37.9	48.2	35.1	52.5	0.0030	<0.0001	<0.0001
	(5.6, 33.6–38.9)	(4.1, 48.7–52.6)	(4.8, 34.3–41.4)	(2.8, 46.1–50.3)	(5.8, 31.5–38.7)	(3.9, 50.1–54.9)			

*Note*: Data are reported as mean (SD, 95% confidence interval) unless otherwise indicated.

Abbreviation: AP, anteroposterior.

The relative femur‐tibia position in the anterior stress radiographs at 90° was significantly improved postoperatively in both groups (*p* < 0.0335) (Table 3). The relative positions in Groups S and D showed no significant differences in comparison with that in the uninjured knee (mean; 62.1%).

**Figure 5:**
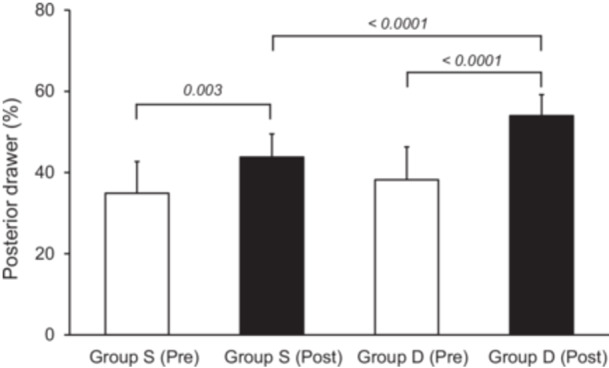
The relative femur‐tibia position in the posterior stress radiographs at 90° of flexion. Group S, single‐bundle posterior cruciate ligament (PCL) reconstruction group; Group D, double‐bundle PCL reconstruction group.

The relative femur‐tibia position in the posterior stress radiographs at 90° was significantly improved postoperatively in both groups (*p* < 0.003) (Table 3) (Figure 5). The relative positions in Group D showed no significant differences in comparison with that in the uninjured knee (mean; 56.8%). Group D was significantly less in the posterior translation than Group S (*p* < 0.0001) (Figure 5). Regarding the isolated PCL injuries, postoperative posterior stress radiographs showed that Group D was significantly less in the posterior translation than Group S (*p* < 0.0001) (Table 4).

In the valgus stress test, the preoperative medial joint opening at 20° significantly improved postoperatively in both groups (*p* < 0.0025) (Table 3). The medial joint opening in Groups S and D showed no significant differences in comparison with that in the uninjured knee (mean; 7.0 mm). There were no significant differences in the postoperative medial joint opening between the groups.

In the varus stress test, the preoperative lateral joint opening at 20° significantly improved postoperatively in Group D (*p* = 0.0042) (Table 3). The lateral joint opening in Groups S and D showed no significant differences in comparison with that in the uninjured knee (mean; 8.3 mm). There were no significant differences in the postoperative lateral joint opening between the groups.

### Objective and subjective clinical results

The Lysholm score and objective IKDC evaluation significantly improved postoperatively in both groups (*p* < 0.0001) (Table 5). All the subscales of the KOOS also significantly improved postoperatively in both groups (*p* < 0.0453) without the subscale of pain and ADL in Group D. The postoperative Tegner scale was significantly greater than the preoperative value in either group (*p* < 0.0001). As for the Lysholm score, the IKDC evaluation, all subscales of the KOOS, the Tegner scale, and the isokinetic peak torque of quadriceps and hamstring muscles, there were no significant differences between the groups.

**Table 5:** Objective and Subjective Clinical Results.

	All injured knee		Group S		Group D		P Value		Intragroup comparison
	Pre	Post	Pre	Post	Pre	Post	Pre versus Post	Pre versus Post	
							Group S	Group D	
Loss of ext. > 5°, No.	6	1	2	1	4	0			> 0.9999
Loss of flex. > 15°, No.	8	2	4	1	4	1			> 0.9999
Lysholm score, points	57.0	87.5	55.7	83.3	57.6	90.2	< 0.0001	< 0.0001	0.2563
	(23.8, 50.4–63.5)	(13.0, 84.0–91.1)	(22.0, 46.0–65.3)	(15.0, 76.8–89.9)	(24.7, 49.0–66.3)	(10.7, 86.4–94.0)			
IKDC, No.							< 0.0001	< 0.0001	0.5056
A	0	15	0	4	0	11			
B	0	22	0	10	0	12			
C	17	13	5	4	12	7			
D	34	3	15	2	19	1			
KOOS, points									
Pain	56.6	76.6	52.4	76.1	58.6	77.5	0.0342	0.106	0.7906
	(20.5, 50.9–62.2)	(19.2, 71.4–81.9)	(17.0, 45.0–59.9)	(20.0, 67.3–84.9)	(21.8, 51.0–66.3)	(16.8, 71.5–83.4)			
Symptom	56.4	78.1	53.1	79.7	58.1	77.4	0.0012	0.0453	0.7417
	(20.8, 50.7–62.1)	(16.9, 73.5–82.8)	(18.8, 44.8–61.3)	(16.6, 72.4–86.9)	(21.5, 50.5–65.6)	(16.5, 71.6–83.1)			
ADL	60.9	82.0	57.0	80.5	62.8	84.3	0.0311	0.0578	0.5361
	(22.5, 54.7–67.0)	(16.1, 77.5–86.4)	(21.0, 47.8–66.2)	(17.7, 72.7–88.2)	(23.0, 54.7–70.9)	(13.6, 79.5–89.1)			
Sport/rec	30.4	65.5	26.6	66.4	32.4	65.7	0.0064	0.0104	0.6435
	(30.3, 22.1–38.8)	(25.4, 58.5–72.4)	(24.2, 16.0–37.2)	(22.7, 56.5–76.4)	(32.8, 20.8–43.9)	(26.2, 56.5–74.9)			
QOL	26.0	65.6	17.2	66.8	30.4	62.9	0.0024	0.0024	0.4438
	(23.8, 19.5–32.5)	(21.1, 59.9–71.4)	(10.8, 12.5–21.9)	(23.8, 56.3–77.2)	(27.1, 20.8–39.9)	(19.3, 56.1–69.7)			
Tegner scale, points	1.2	3.9	1.3	3.9	1.2	3.9	< 0.0001	< 0.0001	0.6211
	(0.5, 1.1–1.4)	(0.9, 3.6–4.1)	(0.6, 1.0–1.5)	(1.1, 3.4–4.3)	(0.5, 1.0–1.4)	(0.7, 3.6–4.1)			
Isokinetic peak torque,									
Quad	55.3	82.8	57.2	89.0	54.0	84.4	< 0.0001	< 0.0001	0.4168
	(18.4, 50.3–60.4)	(10.1, 80.1–85.6)	(15.6, 50.4–64.1)	(6.4, 86.2–91.8)	(20.0, 47.0–61.1)	(11.6, 80.3–88.5)			
Ham	56.2	86.4	52.9	82.2	58.5	83.3	< 0.0001	< 0.0001	0.5392
	(15.2, 52.1–60.4)	(10.0, 83.7–89.2)	(13.8, 46.9–58.9)	(8.8, 78.3–86.1)	(15.8, 53.0–64.1)	(10.9, 79.5–87.1)			

Data are reported as mean (SD, 95% confidence interval) unless otherwise indicated. IKDC: International Knee Documentation Committee; A (normal), B (nearly normal), C (nearly abnormal), D (abnormal), KOOS: Knee Injury and Osteoarthritis Outcome Score, Isokinetic peak torque, % of uninjured knee torque

### Intraoperative and postoperative complications

During surgery, there were no serious complications such as iatrogenic cartilage injuries, serious malposition of the tunnels, graft fixation failure, vascular injuries and so on. There were no serious postoperative complications including fractures and deep vein thrombosis in either group. At the final follow‐up, one patient in Group D who had common peroneal nerve palsy showed total functional recovery. One patient who had acute intraarticular infections in Group D was treated by arthroscopic synovectomy within 2 weeks postoperatively, and continuous irrigation treatment was performed without graft removal.

## DISCUSSION

The important findings of the present study were that, first, the postoperative AP translation at 20° and 70° and the relative femur‐tibia position in the posterior stress radiographs at 90° significantly improved postoperatively in both SB and DB PCL reconstruction groups. Second, the postoperative posterior stability at 90° was significantly better in the DB PCL reconstruction group than in the SB reconstruction group not only in the isolated PCL injuries but also in the MLKIs. Although Lysholm score, objective IKDC evaluation, each subscale of the KOOS, Tegner activity scale, and isokinetic peak torque of quadriceps and hamstrings significantly improved after surgery in both groups, there were no significant differences between the two procedures. Thus, the hypothesis from the introduction of the present study was partially supported.

These results showed a strong possibility that DB PCL reconstruction using hamstring tendon hybrid grafts is effective in improving knee stability, although a prospective randomized study is needed to confirm these findings. In the present study, the knee stability in DB PCL reconstruction group did not result in significantly better results on objective and subjective clinical evaluations. However, this does not mean that the improvement in knee stability was meaningless for the results of DB PCL reconstruction procedure. One of the final goals of PCL reconstruction is the complete restoration of normal knee stability in all patients. Winkler et al. [24] reported that the posterior knee stability affects meniscus damage and/or osteoarthritic changes, resulting in possible superiority in future subjective and functional evaluations. The authors believe that all of the patients who underwent PCL reconstruction simply hoped to achieve the same stability and functionality as in their contralateral knee. It is considered that the ideal goal of PCL reconstruction is to simultaneously restore normal knee stability and normal knee function. From this viewpoint, DB procedure using hamstring tendon hybrid grafts can bring the clinical results of PCL reconstruction closer to the ideal goal.

The PCL complex is divided into the AL bundle, the PM bundle, and the meniscal femoral ligaments [22]. While previously it was assumed that the two bundles function independently, recent studies have demonstrated that the two bundles function synergistically and co‐dominantly, with the AL bundle tensioning during knee flexion and the PM bundle tensioning during knee extension [22]. The AL bundle and PM bundle function synergistically throughout knee motion to resist against posterior tibial translation and internal/external rotation beyond 90° of flexion. Recent biomechanical studies have consistently found superior outcomes with the DB PCL reconstructive technique relative to SB techniques [19] Wijdicks et al. [22] reported that there is increased internal and external rotation following SB PCL reconstruction compared to an intact PCL. These findings suggest that SB PCL reconstruction is not sufficient at restoring knee kinematics and stability throughout all ranges of motion and therefore support the use of a DB PCL reconstruction.

SB versus DB reconstruction procedures have been compared in recent studies [8, 22, 26]. Many investigators demonstrated that clinical results after DB PCL reconstruction with ‘one tibial tunnel’ were compared with those after SB reconstruction. Yoon et al. [26] reported that the DB reconstruction using the Achilles allograft showed better results in posterior stability and IKDC examination than the SB reconstruction did. However, there were no significant differences between SB and DB PCL reconstructions in clinical and radiologic outcomes at a minimum follow‐up of 10 years. Jain et al. [8] analysed the clinical and functional outcome after both SB and DB PCL reconstructions using autologous hamstring grafts. Though DB PCL reconstruction results in less posterior laxity, there is no difference in functional outcome of SB and DB reconstructions. Guo et al. [4] reported that the posterior translation and the rotational angle of the 4‐tunnel DB PCL reconstruction group were significantly lower than that of 3‐tunnel DB group and the SB group. No statistical difference was found between the 4‐tunnel DB group and the intact knee group concerning the posterior tibia translation, the rotational angle, and the valgus–varus‐angle. Li et al. [15] reported that the 4‐tunnel DB technique using tibialis anterior allografts brings about better posterior stability along with the objective and subjective IKDC evaluations compared with the SB technique. In the present study, 4‐tunnel DB PCL reconstruction was significantly less in posterior translation stability than 2‐tunnel SB reconstruction.

In the present study, the hamstring tendon autografts were selected for PCL reconstruction. However, it is well known that weak points of the hamstring tendon graft fixed with sutures to the bone are (1) low stiffness of the graft‐suture‐bone complex, (2) rapid relaxation of the graft tension after surgery, and (3) difficulty in tension control during graft fixation. The hamstring ‘hybrid’ graft was used to improve upon these weak points. Namely, the femur graft‐tibia complex with the hybrid graft involves the following advantages according to biomechanical properties with the tensile test and the cyclic loading test [6, 12, 25], (1) higher stiffness and stronger ultimate load than the complex with the suture method; (2) more resistance to the graft tension relaxation; and (3) clinically, an acceptably long and thick hybrid graft can be fashioned by surgeons with a relatively short or thin autogenous tendon, and the hybrid graft can be more easily fixed to the bone, applying a tension quantified by using a tensiometer to the graft. However, one of the critical issues when using the autograft is potential graft site morbidity as more than two graft constructs are needed in combined ligament reconstruction. Although we could safely use multiple grafts in the MLKIs according to the algorithm as shown in Table 1, we should be very careful not to cause the postoperative functional disability of the knee. Objective and subjective clinical outcomes of the present study were comparable to previous studies [2, 3, 14]. The present study showed the effectiveness of PCL reconstruction using hamstring tendon ‘hybrid’ autografts in isolated PCL injury and MLKIs that can adequately restore satisfactory stability.

Concerning PCL deficient knees including the MLKIs, Wiley et al. [23] reported that the laxity profile of the reconstructed combined PCL/PLC–sectioned knee was better with the tested DB reconstruction technique than with the tested SB reconstruction technique. These data suggest that a DB reconstruction can more closely restore the biomechanics of the intact knee than the SB reconstruction. However, Kim et al. [11] reported that DB PCL reconstruction combined with PLC reconstruction did not appear to have advantages over SB PCL reconstruction combined with PLC reconstruction with respect to the clinical outcomes or posterior knee stability. Hatayama et al. [5] reported functional clinical outcomes determined using the objective IKDC grade and subjective Tegner activity scale had a tendency to be better after DB PCL reconstruction, but there was no significant difference in short‐term stability between the SB and DB reconstructions using hamstring grafts. Regarding systematic review and meta‐analysis, Chahla et al. [2] reported improvements from baseline in subjective clinical outcomes and knee stability in both SB an DB PCL reconstructions. Objective posterior tibial translation measured with a Telos device was found to be significantly lower for the DB group than the SB group. This is in agreement with biomechanical findings that demonstrated a DB PCL reconstruction is superior to a SB PCL reconstruction for restoring native knee kinematics [23]. Krott et al. [14] performed a systematic review and compared the functional and objective outcomes after SB versus DB PCL reconstruction. The only significant difference favoring DB over SB was found in terms of objective clinical outcomes for posterior stability when measured with Telos stress radiography. However, recently, Dasari et al. [3] performed a meta‐analysis of comparative clinical and biomechanical studies to differentiate the pooled outcomes of SB and DB PCL reconstruction cohorts in isolated PCL injury or MLKIs. They concluded that DB PCL reconstruction leads to superior biomechanical outcomes and clinical outcomes relative to SB PCL reconstruction. There were no significant differences between the groups for Lysholm scores, Tegner scales, or risk for a major complication. The results of the present study support this meta‐analysis. 4‐tunnel DB PCL reconstruction improved posterior knee stability in both isolated and combined PCL lesions.

These biomechanical and clinical studies support the present results in knee stability after the DB PCL reconstruction in comparison with the SB reconstruction. Namely, external forces loaded to the PCL are distributed to the two reconstructed bundles. Therefore, excessively overloading to 1 bundle can be avoided during the remodeling phase, resulting in good maturation of not only the AL graft but also the PM graft. In addition, because each bundle in the DB PCL reconstruction is thinner than in the SB reconstruction, the core portion of the former graft may be revascularized sooner than that of the latter graft. At the present time, however, we have to state that these theoretical advantages should be proved in future studies.

Technically, however, the DB PCL reconstruction procedure may be more difficult than SB procedures. There has been considerable concern that the complication rate may be higher in the DB reconstruction than for SB procedures. However, the present study showed that there were no significant differences in the intraoperative and postoperative complication rate between the two procedures. This result suggested that the complication rate in the DB PCL reconstruction is the same as that in SB procedures, if experienced surgeons perform this surgery. These clinical results show that the DB reconstruction procedure is not a risky procedure concerning the fundamental clinical measures compared with the SB procedure.

Recently, patient‐reported outcome measures (PROMs) are increasingly common in order to outline the subjective effects of an intervention. Migliorini et al. [18] investigated whether DB PCL reconstruction is superior to SB reconstruction in terms of PROMs and joint stability. The Tegner scale and the Telos stress were more favorable in the DB cohort. Similarity was found in instrumental laxity and Lysholm score. Two of the major thresholds used to determine the clinical relevance of changes in PROMs include minimal clinically important difference (MCID) and the patient acceptable symptom state (PASS). Liu et al. [16] reported that PCL reconstruction is a reliable surgery for middle‑aged patients suffering from persistent instability, with significant improvement in PROMs that exceeded MCID in the majority of patients, and approximately half of the patients returned to preinjury activity levels. In the present study, a ΔLysholm score exceeding 8.9; and a ΔTegner activity scale exceeding 0.5 based on previously published threshold [16]. Therefore, patients receiving SB and DB PCL reconstruction surgery showed significant improvements in the Lysholm score, and Tegner activity level not only in the isolated PCL injury but also in the MLKIs.

There were several limitations in this study. The main limit of this study was a retrospective study. A second limit was the small number of cases. A third limit was the heterogeneity of the cases, which included isolated PCL injury and MLKIs. Fourth, the relative femur‐tibia position in the AP stress radiograph were measured as the percentage. However, more frequently the posterior drawer is measured in mm side‐to‐side difference. Fifth, the present study did not measure external rotations of the knee after surgery. Further long‐term studies are needed to assess the subjective and objective patient outcomes of DB procedure in patients with the PCL‐deficient knee.

## CONCLUSION

First, the postoperative AP translation and the relative femur‐tibia position in the posterior stress radiographs significantly improved postoperatively in both SB and DB PCL reconstruction groups. Second, the postoperative side‐to‐side differences in AP translation at 20° and 70° showed no significant difference between the groups. There were no significant differences in the Lysholm score, the objective IKDC evaluation, and the KOOS, the Tegner activity scale between both groups although there was significantly better posterior stability in 90° flexion with DB reconstruction.

## AUTHOR CONTRIBUTIONS

Eiji Kondo and Yoshio Nishida designed the study. Zenta Joutoku, Koji Iwasaki, Tomohiro Onodera and Daisuke Kawamura performed clinical assessments. Daisuke Momma and Tomonori Yagi carried out the statistical analyses. Eiji Kondo, Yoshio Nishida and Zenta Joutoku wrote the manuscript. All authors provided critical feedback and helped shape the manuscript. All authors approved the final manuscript. Kazunori Yasuda and Norimasa Iwasaki supervised the project.

## CONFLICT OF INTEREST STATEMENT

The authors declare no conflict of interest.

## ETHICS STATEMENT

Ethical approval was granted from the ethics committee of Hokkaido University Hospital. Informed consent was obtained from all individual participants included in the study. The research results are published at a general level. It is not possible to identify an individual from the results. The manuscript does not contain data of individuals in any form (including personal data, images or videos).

## Data Availability

Only members of the research group have access to the data. Statistical analysis was performed using statistical software (GraphPad Software).
